# Training-Specific Neural Plasticity in Spinal Reflexes after Incomplete Spinal Cord Injury

**DOI:** 10.1155/2016/6718763

**Published:** 2016-09-20

**Authors:** Atif S. Khan, Susan K. Patrick, Francois D. Roy, Monica A. Gorassini, Jaynie F. Yang

**Affiliations:** ^1^Neuroscience and Mental Health Institute, University of Alberta, Edmonton, AB, Canada; ^2^Faculty of Pharmacy & Pharmaceutical Sciences, University of Alberta, Edmonton, AB, Canada; ^3^Department of Surgery, University of Alberta, Edmonton, AB, Canada; ^4^Department of Biomedical Engineering, University of Alberta, Edmonton, AB, Canada; ^5^Department of Physical Therapy, University of Alberta, Edmonton, AB, Canada

## Abstract

The neural plasticity of spinal reflexes after two contrasting forms of walking training was determined in individuals with chronic, motor-incomplete spinal cord injury (SCI). Endurance Training involved treadmill walking for as long as possible, and Precision Training involved walking precisely over obstacles and onto targets overground. Twenty participants started either Endurance or Precision Training for 2 months and then crossed over after a 2-month rest period to the other form of training for 2 months. Measures were taken before and after each phase of training and rest. The cutaneomuscular reflex (CMR) during walking was evoked in the soleus (SOL) and tibialis anterior muscles by stimulating the posterior tibial nerve at the ankle. Clonus was estimated from the EMG power in the SOL during unperturbed walking. The inhibitory component of the SOL CMR was enhanced after Endurance but not Precision Training. Clonus did not change after either form of training. Participants with lower reflex excitability tended to be better walkers (i.e., faster walking speeds) prior to training, and the reduction in clonus was significantly correlated with the improvement in walking speed and distance. Thus, reflex excitability responded in a training-specific way, with the reduction in reflex excitability related to improvements in walking function. Trial registration number is NCT01765153.

## 1. Introduction

Intensive motor training induces task-specific changes in the excitability of spinal reflexes in people without injuries to the central nervous system [1, 2]. For example, resistance training of the plantarflexor muscles over many weeks increases the excitability of the soleus (SOL) Hoffmann (H) reflex [3, 4], whereas balance training on unstable surfaces for a few weeks does the opposite [5–7]. Furthermore, professional athletes who spend years training intensively in their sport show sport-specific reflex excitability. For example, ballet dancers and athletes training in explosive leg sports (e.g., sprinting) show small H-reflexes and tendon reflexes in the SOL [8–10], whereas athletes training in endurance sports, such as middle and long distance running, show large H-reflexes [11]. Thus, motor training alters reflexes in a task-specific way.

Individuals with spinal cord injury (SCI) can exhibit abnormally exaggerated reflex excitability, for example, in the tendon or H-reflex of the soleus (SOL) [12] and in the cutaneous or cutaneomuscular reflex elicited by stimulation of peripheral nerves at the ankle [13]. Whether this increased reflex excitability is useful for, or detrimental to, functional movements remains uncertain [14, 15]. Some suggest that exaggerated reflex excitability might be useful because it can augment hand grip, aid movements such as transfers, and help maintain muscle mass and strength [16], although this is largely based on expert opinion [15, 17].

As with uninjured individuals, intensive motor training after SCI, such as walking on a treadmill, is associated with changes in spinal reflexes such as reduced clonus [18, 19], stretch reflex amplitude [20], and flexor reflex excitability [21, 22]. It remains to be determined if these individuals also show training-specific spinal plasticity in response to different forms of exercises.

We studied two forms of walking training in people with incomplete SCI, one emphasizing walking for as long and as fast as possible on a treadmill, Endurance Training, and the other emphasizing stepping over obstacles and precisely onto targets overground, Precision Training. We previously reported functional improvements in both walking endurance and skill [23] and strengthening of the corticospinal pathways to the tibialis anterior (TA) motoneurons [24] after both forms of training.

Here, we report the neural plasticity induced in spinal circuits from cutaneomuscular afferents in the foot to the SOL motoneurons and their antagonist, the TA, in the same individuals but during the functional task of walking. We examined two reflexes during treadmill walking: the cutaneomuscular reflex (CMR), induced by stimulating the posterior tibial nerve (PTN) [25, 26], and clonus, induced by the natural recurrent activation of monosynaptic Ia inputs by the walking [27, 28]. Portions of this data have been presented in abstract form [29].

## 2. Materials and Methods

### 2.1. Participants

Participants were recruited through a website (http://www.scialberta.ca/), through clinicians and from personal contact in the community.* Inclusion criteria* include nonprogressive SCI between C1–L1 neurological levels acquired ≥7 months before enrolment, with the ability to provide informed consent, walk independently for ≥5 meters using walking aids and/or braces, and attend training sessions 5 days/week.* Exclusion criteria* include severe head injury, cognitive impairment, or other comorbidities that preclude participation in an intensive training program or transcranial magnetic stimulation (TMS, one of the outcome measures of the training [24]).

Ethical approval was provided by the Health Research Ethics Board at the University of Alberta and Alberta Health Services, Pro00003873. Written consent was obtained from all participants.

### 2.2. Experimental Design

**Figure 1:**
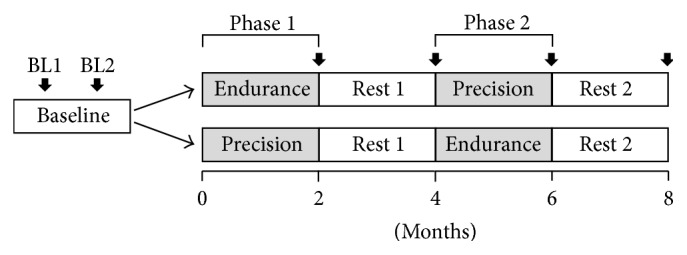
Experimental design. Training consisted of two months of each of Endurance and Precision Training, in a crossover design with rest periods interspersed. Participants were randomly allocated to start with either Endurance or Precision Training. Reflex and walking measures were taken twice at baseline (BL; ~1 week apart) and after each training and rest period (solid vertical arrows).

Participants were block randomized (block size = 4) to begin Endurance or Precision Training for two months (Phase 1), followed by two months of rest (Rest 1), and then crossed over to the other exercise regimen for another two months of training (Phase 2), followed by another 2 months of rest (Rest 2; Figure 1). Reflexes were measured twice at baseline (~1 week apart) and once after each period of training and rest (see Section 2.4 *Measurements*). Overground walking speed, distance, skill, and spinal reflexes were measured at the same time points (at black arrows in Figure 1).

### 2.3. Training

Target training frequency was ~1 hour/day, 3–5 days/week. Details of the training procedures, published previously [23], are briefly described below.

#### 2.3.1. Precision Training

Participants trained to step over obstacles without touching them and onto circular targets by obscuring the whole target with the foot, along a 15 m straight hallway with ~15 obstacles and targets spaced ~1 m apart. The difficulty of the initial training course was determined by trial and error, and it varied daily based on errors made on the previous day, aiming for an error rate of 20%. An error was defined as contact with an obstacle or failing to obscure a target completely. With improvement, the difficulty was increased by reducing support provided by walking aids, increasing obstacle height and width, increasing the size of the circular targets (making it more difficult to completely cover the target), and disallowing extra steps between obstacles and targets. Not all participants reached this last level of difficulty.

#### 2.3.2. Endurance Training

Participants were trained to walk for as long and as fast as possible on a level treadmill with minimal rests. Body-weight support (BWS) and manual stepping assistance were provided only if necessary. The initial treadmill speed was set to be higher than the baseline of overground walking speed (see 10-Meter Walk Test (10MWT) in Table 1). Progression in training is comprised of reducing BWS, manual stepping assistance, and rests, while increasing walking speed and distance.

### 2.4. Measurements

All measures were taken on a nontraining day to minimize the effects of fatigue on the results of reflexes and the walking outcomes.

#### 2.4.1. Instrumentation

Bipolar surface electromyograms (EMG) were recorded from the TA, SOL, and abductor hallucis brevis (AHB) muscles using disposable Ag/AgCl electrodes (Kendall H59P, Mansfield, MA), placed 2 cm apart center-to-center. EMG signals were amplified and band-passed filtered between 10 and 1,000 Hz (AMT-8 EMG System, Bortec Biomedical Ltd., Calgary, AB, Canada). Knee angles were recorded using electrogoniometers bilaterally (Biometrics Ltd., type K100, Newport, UK) in the sagittal plane. All signals were digitized at 5 kHz (Axon Instruments, Digidata 1322A, Union City, CA) and stored for offline analyses.

#### 2.4.2. Cutaneomuscular Reflex and Clonus

Reflexes were measured during walking on a level treadmill, with the speed and BWS being held constant across all testing sessions. The CMR was measured from the SOL and TA muscles in the more spastic leg on the basis of the participant's self-report and opinion of the clinical staff. The CMR was recorded in response to triple pulse stimulation (200 Hz, 0.3 ms pulse width; Digitimer, DS7A, Hertfordshire, England) of the posterior tibial nerve at the level of the medial malleolus, delivered pseudorandomly at intervals between 2 and 5 sec to reduce predictability. The posterior tibial nerve was chosen because (i) it is a mixed nerve that allows recording of an M-wave during movement to ensure that responses are compared at similar effective stimulus intensities (i.e., matched M-wave size) and (ii) stimulus intensity can be compared between days for participants that have impaired sensation. The nerve was first located with a hand-held probe, as we have done in the past [25], and once an optimal location was identified, the probe was replaced with a disposable 1 cm diameter Ag/AgCl adhesive electrode. The anode was a 5 cm × 10 cm disposable adhesive electrode (Axelgaard Manufacturing Co., Fallbrook, CA), placed on the lateral aspect of the ankle joint. Both electrodes were held in place with additional skin tape. Stimuli were applied at a few intensities around 1.5x motor threshold (MT) of the AHB muscle (typically at 1.5x MT and then one intensity above and one below 1.5x MT) to ensure similar stimulus intensities (i.e., matched M-waves) throughout walking and across different experiment days (see* Data Analyses*), as done in the past [25, 26]. The intensity of 1.5x MT was chosen to be consistent with previous reports of this reflex [25]. Motor threshold, estimated in standing, was defined as the stimulus intensity eliciting an M-wave with 50% probability. A minimum of 3 to 4 trials (~3 min each) were required in order to provide a sufficient number of sweeps to match the M-wave size during the walking cycle.

A single trial of ~3 min walking without stimulation (i.e., unperturbed walking) was also recorded. EMG from this control trial was subtracted from the EMG trace obtained during the stimulation trials to reveal the reflex response. The SOL EMG during the entire length of the unperturbed walking trial was also used to estimate clonus ([19]; see* Data Analyses*).

#### 2.4.3. Over Ground Walking

Self-selected walking speed was estimated using the 10MWT [30] (time to walk the middle 10 m of a 14 m straight track). The 6-Minute Walk test (6MWT) measured the maximum distance covered while walking back and forth along a straight 30 m track for 6 min [31]. The Spinal Cord Injury-Functional Ambulation Profile (SCI-FAP), which contains 7 timed tasks during walking (carpet, up-and-go, walking over and around obstacles, up and down stairs, up and down a curb, walking while carrying a bag, and walking while opening door), measured walking skill [32]. All measures have been validated for people with SCI [30, 33].

### 2.5. Data Analyses

#### 2.5.1. Cutaneomuscular Reflex

Reflexes were analyzed using custom-written MatLab programs (The MathWorks, Inc., Natick, MA). The EMG responses were sorted according to the time of occurrence of the stimulus within the walking stride. The beginning of a walking cycle was set to be near mid-swing on the stimulated side, based on a threshold crossing of the knee goniometer signal. Because the duration of each stride can vary, the strides were first normalized in time from the beginning of one cycle to the beginning of the next cycle and then divided into 8 bins of equal duration, using the MatLab software. Reflexes elicited with AHB M-wave amplitudes (peak-to-peak) within a target range were analyzed to control for stimulus intensity during movement, as pioneered by Capaday and Stein (1986) [34]. The target range was selected after examining the distribution of M-wave amplitudes for each participant before and after training, with the range being restricted to differences in the upper and lower limits of <20% of the maximum M-wave (M_max_), consistent with previous reports [25]. Reflex responses in each bin were full-wave rectified, low-pass filtered at 200 Hz (dual-pass, zero phase, 2nd-order, digital Butterworth filter), time-locked to the stimuli, and averaged.

**Figure 2:**
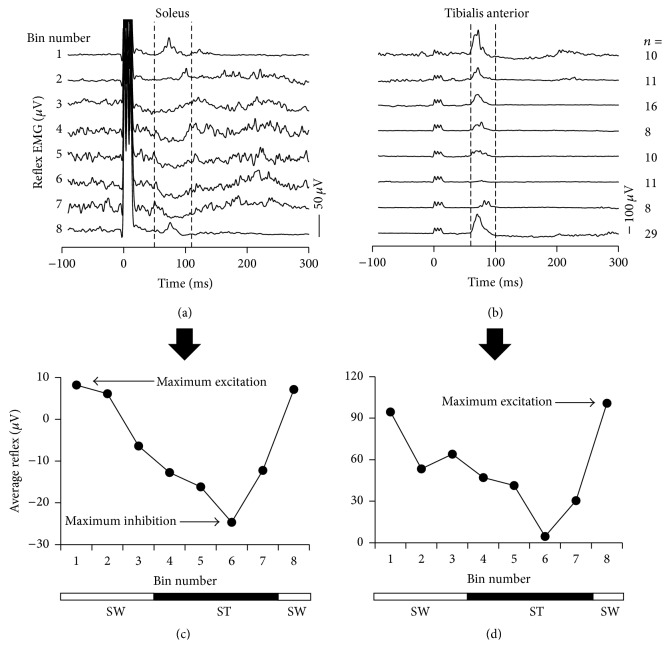
The cutaneomuscular reflex (CMR) during walking in a single participant. Average EMG traces from the soleus (a) and tibialis anterior (b) muscles, time-locked to the beginning of the stimulus (time = 0), are shown for the 8 bins of the walking cycle from participant P12 after subtraction of the background EMG during unperturbed walking. The number of acceptable reflexes within each time bin (bin number) across the step cycle is indicated on the far right. Data were obtained after the first rest period (i.e., at Month 4) prior to Endurance Training. Triple pulses (200 Hz; 0.3 ms pulse width; at ~1.5x motor threshold for the abductor hallucis brevis (AHB) muscle) were applied to the posterior tibial nerve behind the medial malleolus during walking on a treadmill. Responses displayed are matched for M-wave amplitudes, measured from the AHB (see* Data Analyses* for details). Responses for each of the 8 bins of the walking cycle were quantified by averaging the EMG between vertical dashed lines in (a) and (b) and plotted as a function of time in the walking cycle in (c) and (d). Correspondences of the bin numbers with stance (ST) and swing (SW) phases are shown by the solid and open bars, respectively, at the bottom of the graphs. Horizontal arrows in (c) and (d) indicate maximum inhibition and excitation.

**Figure 3:**
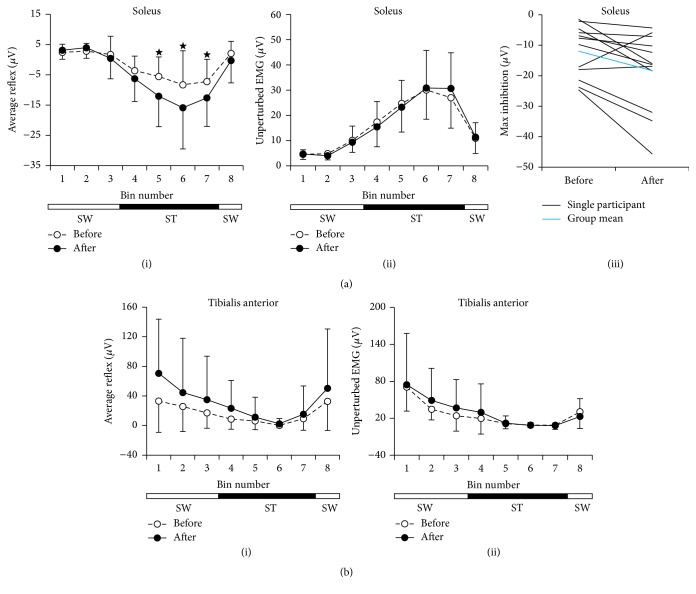
Changes in reflex excitability during walking varied with training type. The cutaneomuscular reflex (CMR) during 8 bins in the walking cycle, averaged across participants (*n* = 12), are shown for the soleus (a)(i) and tibialis anterior (b)(i) muscles before and after Endurance Training. Significant differences in post hoc comparisons are indicated with ★. Average EMG corresponding to the bin numbers during unperturbed walking trials are shown in (a)(ii) and (b)(ii). Error bars represent one standard deviation. ST: stance phase (solid bars); SW: swing phase (open bars). (a)(iii) Maximum (Max) inhibition of the SOL CMR before and after Endurance Training is shown for each participant, with the group mean superimposed.

EMG traces during unperturbed walking were similarly divided into 8 equal bins and subtracted from EMG responses with stimulation to reveal the reflex response [25, 26]. All acceptable reflexes were analyzed, unless the subtraction was deemed unsatisfactory by visual inspection, revealed by a nonzero baseline prior to the stimulus. This ensured that the levels of EMG were matched across the control and stimulated conditions. The start and end times (i.e., window) of reflex responses were determined by visually examining the response across all 8 bins separately for each participant (e.g., Figures 2(a) and 2(b), vertical dashed lines) and kept consistent before and after training. The reflex response was the averaged EMG amplitude over the duration of the window for each bin (e.g., Figures 2(a) and 2(c) (SOL) and 2(b) and 2(d) (TA)) within a participant. These bin averages were then averaged bin-by-bin across participants (e.g., Figures 3(a)(i) and 3(b)(i)) to reveal the pattern of reflex modulation of the CMR over the step cycle. Reflex inhibition and excitation in the step cycle were also quantified using the maximum inhibition (for SOL only, e.g., Figure 2(c)) and excitation (for TA and SOL, e.g., Figures 2(c) and 2(d)) of the CMR, wherever it happened in the step cycle and used to correlate with the walking outcomes. Since there were both excitation and inhibition in the SOL through the cycle but only excitation in the TA, only these were quantified. As the walking outcomes have been reported previously [23], those analyses are not repeated here.

#### 2.5.2. Clonus

The raw SOL EMG signal was corrected for the DC offset, rectified, and analyzed in the frequency domain using fast Fourier transform within the clonus frequency range set to 4–10 Hz in agreement with other studies [19, 27, 28, 35, 36]. To normalize the amplitude of the clonic EMG bursts to that of the regular EMG during walking, the 4–10 Hz signal power was expressed as a fraction of the total power within 0–40 Hz, according to clonus power (power between 4–10 Hz)/(power between 0–40 Hz). Frequencies > 40 Hz were excluded to avoid power-line noise. Clonus power from the more spastic side, that is, the side with the higher average clonus power across the baseline measures, was used in the analyses.

### 2.6. Statistical Analyses

#### 2.6.1. Were the Two Baseline Measures Repeatable?

The two baseline measures of reflexes taken approximately 1 week apart (i.e., BL1 and BL2 in Figure 1) were compared for all participants, regardless of which training they did first, to determine repeatability using the Paired Samples *t*-test (or Wilcoxon Signed Rank test for data that was not normally distributed). Baseline comparisons were made for the maximum inhibition, maximum excitation, and clonus, and a two-way repeated-measures ANOVA for the reflex modulation of the CMR (factor 1: time across the step cycle (i.e., bins 1–8); factor 2: time of measurement of the baseline measures (i.e., Baseline 1 and Baseline 2)). In cases where the two baseline measures were not statistically different (all CMR data), the two baseline measures were averaged to provide the best estimate for pretraining conditions. Participant characteristics (age, time since injury, 10MWT, 6MWT, and SCI-FAP) at baseline were also compared between the 2 groups (Precision or Endurance first) using an Independent Sample *t*-test (or Mann–Whitney *U* test).

#### 2.6.2. Did the Two Types of Training Affect Reflex Excitability in Different Ways?

*(1) CMR Modulation in Walking*. The CMR was quantified for before and after each type of training separately, regardless of when the training occurred (i.e., Phase 1 or 2). For example, for those who had Endurance Training in Phase 1, before and after training measures were those taken at baseline and at the end of Phase 1 (i.e., at end of Month 2; see Figure 1), respectively. For those who had Endurance Training in Phase 2, before and after training measures were those taken at the end of Rest 1 (i.e., end of Month 4) and at end of Phase 2 (i.e., end of Month 6), respectively. The CMR for each of the two groups before and after Endurance Training was then pooled across phase (i.e., Phase 1 and Phase 2) to counterbalance the possible effect of the order of training (Figure 1, see also [23]). The same was done for Precision Training.

The distribution of the data was tested using the Shapiro-Wilk test for normality. To determine the effects of training on reflex excitability, the modulation of the CMR across the step cycle was compared using a 2-way repeated-measures ANOVA, with treatment effect (i.e., before and after) and time bin of the step cycle (i.e., bins 1–8) as the two factors. Missing data in the ANOVA, such as when data was excluded at certain bins due to unsatisfactory subtraction of the background EMG (see* Data Analyses*), was managed by replacing the missing value with the average reflex value for the respective bins. A Greenhouse-Geisser correction was applied if the assumption of sphericity was not satisfied (i.e., *p* < 0.05 on Mauchly's test of sphericity) or if the test was not performed due to insufficient degrees of freedom as a result of a small sample size. Post hoc analysis of significant findings was performed using Tukey's Honestly Significant Difference test.

To ensure that the results were not distorted by the crossover design, two further analyses were performed. First, the changes as a result of Precision or Endurance Training were compared for Phase 1 only, using the same statistical procedures (as above). Second, to determine if the rest period between Phases 1 and 2 resulted in changes to the reflexes, a similar 2-way repeated-measures ANOVA was used to compare the measures at the beginning and at end of Rest 1 (i.e., at the end of Month 2 versus Month 4, Figure 1). To be complete, the comparisons were also made for Rest 2 (i.e., at the end of Month 6 versus Month 8).

*(2) Clonus Power*. Clonus was higher at Baseline 1 compared to Baseline 2 (Baseline 1: 0.13 ± 0.08, and Baseline 2: 0.10 ± 0.07; paired *t*-test: *p* = 0.031), which could be related to familiarity with the testing environment, since nervousness during Baseline 1 could have caused greater reflex excitability [37]. Hence, we used clonus power at Baseline 2 as the more conservative estimate for clonus before Phase 1. Otherwise, comparisons were made for clonus measured before and after each form of training and collapsed across phase as described for the CMR (above). Unlike the CMR modulation, there was only one measure taken before and after each type of training, so a Paired Samples *t*-test (or Wilcoxon Signed Rank test) was used to determine if there was a significant change as a result of the training.

#### 2.6.3. Was the Reflex Excitability Related to Walking Ability?

The relationship between reflex excitability and walking ability was quantified with linear regression. The maximum inhibition of the CMR during the walking cycle and the clonus power were each examined as a function of the 3 walking measures (10MWT, 6MWT, and SCI-FAP) prior to the start of any training. Since there were strong trends, including significant relationships between clonus and walking ability, we further determined if the overall change in clonus (i.e., from Baseline 2 to the end of Month 6) was related to the overall change in walking ability. The relationship between maximum inhibition of the SOL CMR and walking ability was similarly examined for before Endurance Training, since only Endurance Training induced a significant change in the CMR inhibition (see below). The relationship between change in the maximum inhibition of the SOL CMR and change in walking ability was also examined. Significance for all tests was set to *p* < 0.05.

## 3. Results

### 3.1. Participants

Of the original 20 participants who contributed data to the previous paper [23], all completed Precision Training and 17 completed Endurance Training. One participant (P15) was excluded from the current paper because he had no muscle activity in the TA and SOL (lesion level L1). Participant characteristics are shown in Table 1, with the order of training indicated; no differences in participant characteristics were observed at baseline between the two groups. To facilitate comparison with our previous publication [23], participants are described using the same codes.

For measures of clonus, all 19 participants were included in our analysis of Precision Training, and 14 for Endurance Training. Two participants were excluded from analysis of Endurance Training because one had large single motor unit activity in the SOL during walking (P4), whose firing rates confounded the clonus estimate [38], and the other had a technical problem with the EMG recording (P18).

For the CMR, 14 data sets were included for Precision Training and 12 for Endurance Training. Three participants were excluded from the analysis of both forms of training because the stimuli either stopped their walking (P6, P18) or induced extensive clonus (P8), making it impossible to collect this data. P4 was additionally excluded from analysis of Endurance Training due to inadequate number of responses with acceptable M-wave matches. Two additional participants were excluded from the analysis of Precision Training due to extensive clonus generated by the stimuli (P3), or poor subtraction of unperturbed EMG, resulting in uncertainty of the CMR response (P12).

The walking speeds during reflex testing ranged from 0.13 to 0.89 m/s (mean ± SD = 0.35 ± 0.18 m/s). Body-weight support was used by 6 participants, which ranged from 9 to 27 kg (mean ± SD = 20 ± 7 kg).

### 3.2. Baseline Measures

The pattern of reflex modulation of the CMR across the step cycle did not differ between the two baselines (2-way repeated-measures ANOVA (factor 1: bin number; factor 2: baseline measure) and ANOVA interaction effect: SOL: *p* = 0.84 and TA: *p* = 0.3), neither did the maximum inhibition (Baseline 1: −15.5 ± 18.3 *μ*V and Baseline 2: −16.3 ± 14.5 *μ*V; paired *t*-test: *p* = 0.71) and excitation (Baseline 1: 7.4 ± 6.2 *μ*V and Baseline 2: 6.4 ± 6.0 *μ*V; paired *t*-test: *p* = 0.76) of the SOL CMR and the maximum excitation of the TA CMR (Baseline 1: 33.2 ± 24.6 *μ*V and Baseline 2: 25.2 ± 27.5 *μ*V; paired *t*-test: *p* = 0.31). Clonus power at Baseline 1 was higher than at Baseline 2 (Baseline 1: 0.13 ± 0.08 and Baseline 2: 0.10 ± 0.07; paired *t*-test: *p* = 0.031), so Baseline 2 was used to represent pretraining values for Phase 1 (see* Statistical Analysis* for rationale).

### 3.3. Cutaneomuscular Reflexes

Representative data from participant P12 is shown in Figures 2(a) and 2(b), illustrating reflex modulation across a walking cycle for the SOL and TA muscles, respectively. Vertical dashed lines show the time window of the reflex responses used for averaging for this participant, with the averaged responses plotted in Figures 2(c) and 2(d). Average reflex window onset and offset times across participants were 44.7 ± 6 to 107.9 ± 29.4 ms for the SOL and 48.4 ± 8.1 to 101.3 ± 15.9 ms for the TA. Reflex responses during walking in the SOL muscle were dominated by inhibition while those in the TA were dominated by excitation. For this participant, the maximum excitation and inhibition of the SOL CMR occurred in bins 1 and 6, respectively, and maximum excitation of the TA CMR in bin 8.

The repeated-measures ANOVA comparing group data of the reflex amplitude in the SOL before and after Endurance Training (Figure 3(a)(i)) indicated a significant interaction (*p* = 0.047) between treatment effect and time bin of the step cycle and a significant training effect (*p* = 0.011). Post hoc analysis revealed significant increase in inhibition at bins 5 (*p* = 0.014), 6 (*p* = 0.0048), and 7 (*p* = 0.044), resulting in a deeper reflex modulation after training. EMG amplitude during unperturbed walking did not change in the SOL after Endurance Training (ANOVA: interaction effect, *p* = 0.5; training effect, *p* = 1; Figure 3(a)(ii)), suggesting that the greater inhibition is not due to changes in the background muscle excitability. Modulation of reflex excitation in the TA showed no significant changes after Endurance Training (ANOVA: interaction effect, *p* = 0.075; training effect, *p* = 0.082; Figure 3(b)(i)), as with the background EMG during unperturbed walking (ANOVA: interaction effect, *p* = 0.36; training effect, *p* = 0.53; Figure 3(b)(ii)). Precision Training had no effect on the CMR and background EMG activity in either muscle (ANOVA, data not shown). Changes in individual participants before and after Endurance Training are shown in Figure 3(a)(iii). With 2 exceptions, the reflex inhibition of the SOL CMR was enhanced with training.

To determine if there was washout during the rest periods, we compared the modulation of the SOL CMR at the beginning and end of each rest period for Endurance and Precision Training, separately. No differences were found in the 2-way repeated-measures ANOVA (Rest 1: no interaction for Endurance *p* = 0.38 and Precision *p* = 0.29; Rest 2: no interaction for Endurance *p* = 0.25 and Precision *p* = 0.47). Since there was no washout, the residual effects from the first phase of training could have affected the second phase of training. Hence, we reanalyzed the reflex modulation in the SOL separately using Phase 1 data only (i.e., *n* = 6  and 7 for Endurance and Precision Training, resp.). The same trends were observed for the two forms of training: greater inhibition after Endurance Training (ANOVA: interaction effect, *p* = 0.09) and no change after Precision Training (ANOVA: interaction effect, *p* = 0.61).

### 3.4. Clonus

**Figure 4:**
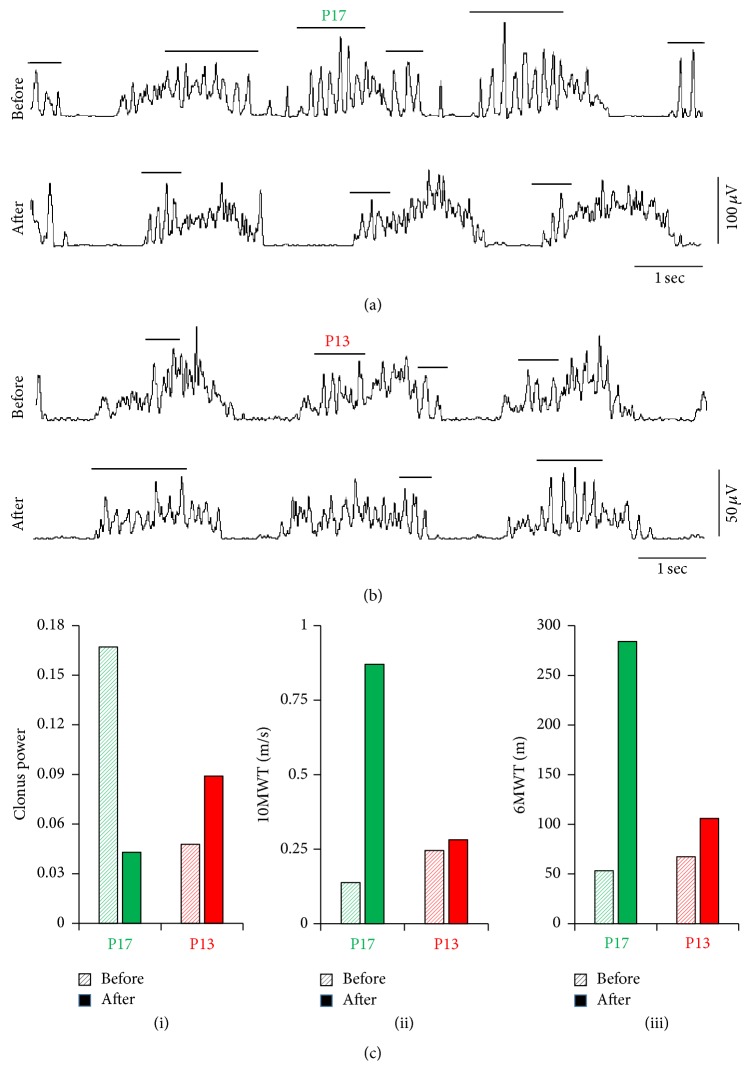
Clonus in the soleus muscle during unperturbed walking. (a) and (b) Rectified and smoothed (dual-pass, zero phase, 20 Hz low-pass, 2nd-order, digital Butterworth filter, for display purposes) EMG traces from the soleus muscle during unperturbed walking on a level treadmill, recorded before (i.e., at Baseline 2) and after both phases of training (i.e., at Month 6), from participants exhibiting (a) decrease and (b) a small increase in clonus after the training. (c)(i) The change in clonus over both phases of training (i.e., change from Baseline 2 to end of Month 6) and the corresponding changes in (ii) the 10-Meter Walk Test (10MWT) and (iii) 6-Minute Walk Test (6MWT) for both participants are shown for comparison. Horizontal lines indicate clonic EMG bursts.

Clonus power changed in different ways for different participants, but those who showed a reduction in clonus also showed an improvement in walking function. Figures 4(a) and 4(b) illustrate rectified, smoothed EMG traces from the SOL muscle during unperturbed walking on a treadmill, from participants exhibiting a decrease (Figure 4(a)) and a small increase (Figure 4(b)) in clonus with training, with large and small increases in walking function (i.e., speed and distance, resp.). Since there were no differences in the way clonus responded to the 2 forms of training across participants, we collapsed the data across training type and considered the change in clonus for each participant from Baseline 2 to the end of training in Phase 2 (i.e., end of Month 6). Changes in clonus power for P17 and P13 are shown in Figure 4(c)(i), with changes in walking ability shown in Figures 4(c)(ii) and 4(c)(iii).

### 3.5. Relationship between Reflex Excitability and Walking Measures

**Figure 5:**
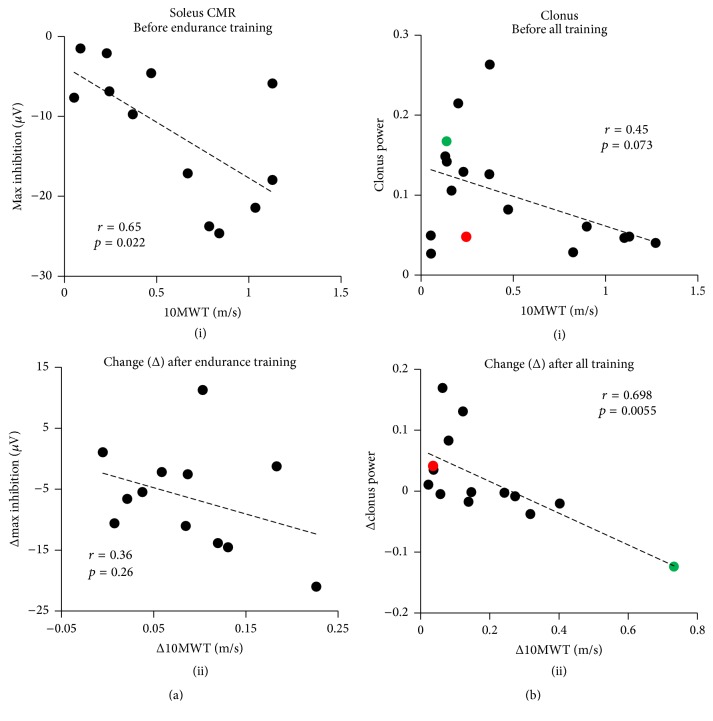
The relationship between reflex excitability and walking outcomes. (a) The inhibition in the soleus CMR was related to walking speed before Endurance Training (i). Change in the maximum inhibition of the CMR as a result of Endurance Training was weakly related to the improvement in walking speed (ii). (b) Clonus in the soleus was weakly related to walking speed before any form of training (i.e., at baseline) (i). Reduction in clonus was significantly related to improvement in walking speed (ii). Green and red data points correspond to P17 and P13, respectively, from Figure 4.

The relationship between the SOL CMR and walking measures was examined for Endurance Training, since only Endurance Training induced a significant enhancement of the inhibition in the SOL CMR. Prior to the start of Endurance Training, there was a relationship between the maximum inhibition in the SOL CMR during walking and walking speed (10MWT, Figure 5(a)(i); *r* = 0.65; *p* = 0.022) and walking distance (6MWT, not shown; *r* = 0.69; *p* = 0.013) and a weaker relationship with walking skill (SCI-FAP, not shown; *r* = 0.53; *p* = 0.078). Improved walking speed as a result of Endurance Training (i.e., Δ10MWT) was weakly associated with enhanced inhibition in the SOL CMR (i.e., Δmax inhibition), but the correlation was not significant (Figure 5(a)(ii); *r* = 0.36; *p* = 0.26). No relationships were observed between the change in the max inhibition of the CMR and walking distance (Δ6MWT, not shown; *r* = 0.2; *p* = 0.53) and skill (ΔSCI-FAP, not shown; *r* = 0.0095; *p* = 0.98).

There were trends for a relationship between clonus and walking measures prior to any training (10MWT in Figure 5(b)(i), *r* = 0.45, *p* = 0.073; 6MWT, not shown, *r* = 0.43, *p* = 0.088). Again, there was no relationship between clonus and walking skill (SCI-FAP, not shown; *r* = 0.13; *p* = 0.61). The reduction in clonus after all training was significantly related to improvements in walking speed (Figure 5(b)(ii), *r* = 0.698, *p* = 0.006) and walking distance (not shown, *r* = 0.59, *p* = 0.026) but not walking skill (not shown, *r* = 0.065, *p* = 0.82). Due to the possibility of a single outlier in the bottom right corner of Figure 5(b)(ii) dominating the results, regression analyses were also performed without the outlier. There remained a trend in the relationship between change in clonus and change in walking speed (*r* = 0.5, *p* = 0.083), but the relationship between change in clonus and change in walking distance was no longer significant (*r* = 0.29, *p* = 0.34).

## 4. Discussion

The primary new finding is that contrasting forms of walking training have differential effects on the excitability of the CMR. Endurance Training enhanced the reflex inhibition in the SOL during walking, while Precision Training did not. Neither form of training changed clonus in a systematic way. Yet there is a significant relationship between the degree of clonus and walking ability, as well as a relationship between improvement in walking and reduction in clonus. Increase in the CMR inhibition induced by Endurance Training was weakly related to improvement in walking. These results suggest that certain forms of walking training can reduce reflex excitability, and some aspects of the reduced reflex excitability are associated with improved walking function.

### 4.1. Methodological Limitations

As with all exercise interventions, the participants cannot be blinded to the intervention. While we told participants that we did not know which method would be better, the participants could have had their own biases. This may have affected the results but likely would not favor one type of training.

The crossover design could have led to confoundedness in Phase 2 of training, as there was no washout from the training in Phase 1. However, since the trends in the results remained the same when we analyzed Phase 1 alone, we do not think this would have changed our overall findings.

### 4.2. How You Train Makes a Difference

Different forms of training in uninjured individuals have differential effects on the functional outcome of walking and spinal reflex excitability (see* Introduction*). Here, Endurance Training resulted in a small, yet significant, increase in inhibition of the SOL CMR during walking (Figure 3(a)) and improvements in walking speed and distance [23], whereas Precision Training only modestly improved walking [23] and did not change the reflex excitability. Interestingly, both forms of training strengthened the corticospinal tracts (CST), as measured using maximum motor evoked potentials from single-pulse TMS [24]. Thus, neural plasticity was differentially affected at the spinal and cortical levels by the two different forms of training. Interestingly, in these injured individuals, the total duration of training to elicit a change was considerably less than the athletes mentioned in* Introduction*, although similar changes to reflex modulation have been induced in uninjured individuals training to execute unfamiliar tasks such as backward walking over a 10-day period [39].

It might be argued that the change in CMR inhibition in the SOL was relatively minor, that is, about −10 *μ*V on average (Figure 3(a)). However, the typical inhibition seen in uninjured individuals during the stance phase of walking is in the order of −30 *μ*V [26], and the pretraining inhibition in our participants was about −5 *μ*V (Figure 3(a)), so an additional −10 *μ*V is a considerable change in the direction towards the pattern of the uninjured.

The reason that Endurance Training was more effective in reducing spinal reflex excitability overall could have been because of the much greater number of steps executed during training compared to Precision Training (steps/session: Endurance ~1200; Precision ~400), or it could be that the two forms of training induced neural plasticity at different neural pathways. The two forms of training emphasized very different aspects of walking: speed and distance for Endurance Training versus skill and accuracy for Precision Training. Thus, it was not feasible to equate the number of steps executed in these two forms of training, because it would have required over 4 hours a day of Precision Training to obtain the same number of steps. Thus, while we do not know the “ingredient” that caused the reduction in reflex excitability with Endurance Training, we suggest that the more likely explanation is that the volume (i.e., number of steps) of training is especially important to induce plasticity in the reflex pathways, because walking skill improved equally with both forms of training as measured by SCI-FAP [23]. A definitive answer will require studies targeting this question specifically, with perhaps more sensitive measures of walking skill than those contained in SCI-FAP.

Two other studies have compared changes in spinal reflex excitability with different forms of walking training after SCI in humans [18, 40]. The changes in H_max_/M_max_ ratio and clonus in the SOL muscle were compared after 4 types of training: (a) BWS treadmill training (BWSTT) with manual assistance, (b) BWSTT with functional electrical stimulation to the common peroneal nerve, (c) training in the Lokomat, and (d) overground training with functional electrical stimulation to the common peroneal nerve [18]. While the excitability and duration of clonus were reduced after all forms of training, H_max_/M_max_ ratio did not change. Thus, consistent with our study, different spinal reflexes could be differentially responsive to the training. The lack of difference between the different types of training could have been because the 4 training methods were more similar compared to the two in the current study, as all 4 training methods involved continuous stepping, with no differences in walking outcomes between the methods. In a different study, changes in clonus and H_max_/M_max_ ratio were compared after 1 month of BWSTT and tilt table standing [40]. Clonus and flexor spasms were reduced only after the treadmill walking, while extensor spasms were reduced after tilt table standing; no change was observed in H_max_/M_max_ after either form of training [40].

### 4.3. Are Training-Induced Changes in Reflex Behavior Related to Improvements in Walking?

Many reports confirm that walking training changes reflex excitability in people with SCI, but few studies have considered its relationship with improvements in walking. BWS step training induced a reduction in SOL clonus duration and an increase in threshold angle for triggering clonus when measured in sitting [18] and lying supine [40], while Lokomat training induced a deeper modulation of the H-reflex throughout the walking cycle [41] and greater rate-dependent depression [42]. Lokomat training, however, did not change the overground walking function as measured by the 6MWT and the timed-up-and-go test [41], so presumably there was no appreciable relationship between walking function and change in reflex excitability (relationship not reported in [38]). Only one study reported a significant relationship between reduced clonus and improved walking speed with training [18].

Cutaneous and cutaneomuscular reflexes also change with walking training. The CMR in the TA muscle in response to posterior tibial nerve stimulation showed enhanced early excitation and reduced late excitation with 4 weeks of Lokomat training [21]. Improvements were seen in the 10MWT, but the relationship between the electrophysiological and functional changes was not reported. Flexor reflex in the TA in response to sural nerve stimulation was also reduced with Lokomat training, but changes in overground walking ability were not reported [22].

We show here that the amount of clonus in the SOL during walking is weakly related to how well the person walks (i.e., 10MWT and 6MWT) before training. Furthermore, reduction in clonus is correlated with improvement in walking outcomes (Figure 5(b)(ii)). Similarly, the depth of inhibition in the CMR is related to walking speed and distance before Endurance Training, and the increase in CMR inhibition as a result of Endurance Training is weakly related to the improvement in walking speed (Figure 5(a)(ii)). The ability to walk is multifactorial and dependent on factors such as the strength of descending input from the brain, strength and endurance of muscles, ability to balance, and excitability of spinal reflexes. Thus, the correlation between any factor and walking ability may be low. Nevertheless, we believe it is essential to understand which aspects of neural plasticity might underlie functional improvements. Indeed, it is possible that some neural plasticity is unrelated to walking function. Determining the correspondence between change in walking function and change in training-induced neural plasticity is the first step towards improving this understanding.

## 5. Conclusions

Our data, along with other converging evidence, suggests that intensive training of walking reduces the abnormal reflex excitability seen after SCI, for example, reduced excitability of the H-reflex [41, 42], stretch reflex [20, 43], and clonus [18, 40] at the ankle, and the flexor reflex [22] and long-latency cutaneomuscular reflex [21] in the lower leg. This enhanced inhibition is concurrent with training-induced strengthening of corticospinal input to the motoneurons/interneurons [24, 44, 45] and better volitional control of leg muscles [24, 46]. Descending control of spinal circuits, especially from the CST, is well described for humans [47–50] including during voluntary movements [51, 52]. It is likely that walking training strengthens the descending control, including inhibitory control of spinal circuits, which have been weakened by the injury [24].

## Figures and Tables

**Table 1:** Participant characteristics.

Endurance Training in Phase 1							
Participant code-gender	Age (yr)	Neurological level of injury	Cause of injury	Time since injury (yr)	10MWT (m/s)	6MWT (m)	SCI-FAP score
P1-F	49	C6	MVA	2.5	0.05	17	649
P2-M	24	T6	MVA	1	0.37	114	119
P3-M	25	T4	MVA	1.1	0.13	48	235
P4-M	57	C5	MVA	34.9	0.90	298	59
P5-M	48	T12	Fall	0.7	0.23	119	140
P6-M	65	C3	MVA	1.2	0.05	19	864
P7-M	60	C3	Bull attack	0.6	0.47	172	286
P8-M	46	C6	MVA	7.3	0.14	43	308
P9-F	63	C4	MVA	6	1.13	278	10
P10-F	45	T2	Surgical clot	2.3	0.33	115	133

*Mean (SD)*	*48.2 (14.4)*			*5.8 (10.5)*	*0.38 (0.37)*	*122 (101)*	*280 (273)*

Precision Training in Phase 1							
Participant code-gender	Age (yr)	Neurological level of injury	Cause of injury	Time since injury (yr)	10MWT (m/s)	6MWT (m)	SCI-FAP score

P11-M	43	T12	Sport	17.5	1.27	392	10
P12-M	63	C4	Sport	20	0.82	254	31
P13-F	21	C6	MVA	1	0.25	67	262
P14-M	61	C5	Sport	2.4	0.90	292	14
P16-M	34	C4	Gun shot	1.2	1.10	337	8
P17-M	32	T2	Fall	0.8	0.16	53	231
P18-F	41	T12	Infection	3.5	0.17	38	602
P19-F	50	T6	Tumor	1.8	0.20	67	165
P20-M	52	T10	Surgical bleed	1.4	0.37	120	117

*Mean (SD)*	*44.1 (13.8)*			*5.5 (7.6)*	*0.6 (0.4)*	*180 (138)*	*160 (192)*

F: female; M: male; C: cervical; T: thoracic; MVA: motor vehicle accident; 10MWT: 10-Meter Walk Test; 6MWT: 6-Minute Walk Test; SD: standard deviation; SCI-FAP: Spinal Cord Injury-Functional Ambulation Profile.
